# Macrophages and Dendritic Cells Are Not the Major Source of Pro-Inflammatory Cytokines Upon SARS-CoV-2 Infection

**DOI:** 10.3389/fimmu.2021.647824

**Published:** 2021-05-26

**Authors:** Marc A. Niles, Patricia Gogesch, Stefanie Kronhart, Samira Ortega Iannazzo, Georg Kochs, Zoe Waibler, Martina Anzaghe

**Affiliations:** ^1^ Section “Product Testing of Immunological Biomedicines”, Paul-Ehrlich-Institut, Langen, Germany; ^2^ Institute of Virology, Medical Center, Faculty of Medicine, University of Freiburg, Freiburg, Germany

**Keywords:** SARS-CoV-2, COVID-19, dendritic cell, macrophages, interleukin 6, tumor necrosis factor alpha/TNF-alpha, type I interferon, activation status

## Abstract

The exact role of innate immune cells upon infection with severe acute respiratory syndrome coronavirus 2 (SARS-CoV-2) and their contribution to the formation of the corona virus-induced disease (COVID)-19 associated cytokine storm is not yet fully understood. We show that human *in vitro* differentiated myeloid dendritic cells (mDC) as well as M1 and M2 macrophages are susceptible to infection with SARS-CoV-2 but are not productively infected. Furthermore, infected mDC, M1-, and M2 macrophages show only slight changes in their activation status. Surprisingly, none of the infected innate immune cells produced the pro-inflammatory cytokines interleukin (IL)−6, tumor necrosis factor (TNF)-α, or interferon (IFN)−α. Moreover, even in co-infection experiments using different stimuli, as well as non-influenza (non-flu) or influenza A (flu) viruses, only very minor IL-6 production was induced. In summary, we conclude that mDC and macrophages are unlikely the source of the first wave of cytokines upon infection with SARS-CoV-2.

## Introduction

In December 2019, a novel coronavirus, later named severe acute respiratory syndrome coronavirus 2 (SARS-CoV-2), caused an outbreak of respiratory illness in Wuhan, China (1–3). Severe infections can rapidly progress to pneumonia and result in coronavirus disease (COVID-19) (4). Due to the rapidly increasing number of infected patients, the WHO declared SARS-CoV-2 a pandemic on March 11, 2020 (1). As of April 29, 2021, data from Johns Hopkins University indicate over one hundred fifty million infections with SARS-CoV-2 in 192 countries with more than 3.15 million deaths worldwide (5).

SARS-CoV-2 is an enveloped positive-sense single-stranded RNA virus with a genome size of 29.9 kb and belongs, like SARS-CoV and Middle East Respiratory Syndrome (MERS), to the beta-coronaviruses. SARS-CoV-2 shares the same entry receptor, Angiotensin-converting enzyme 2 (ACE2), with SARS-CoV which is expressed in type II alveolar epithelial cells (AT2), endothelial cells, fibroblasts, and human airway epithelia (6–8). Other studies predicted an interaction of SARS-CoV-2 spike protein with CD147 and CD26, which are also expressed on innate immune cells (9–11).

SARS-CoV-2 primarily infects pneumocytes and macrophages (12). Infection correlates with an extensive infiltration of neutrophils and macrophages into the lungs. High SARS-CoV-2 viral load is strongly associated with a cytokine storm, which is therefore a marker to predict poor prognosis for the disease. In critically ill COVID-19 patients, levels of interleukin (IL)-6, which is known to be one of the main pro-inflammatory cytokines in the initiation or amplification of a cytokine storm, have been shown to be significantly elevated (3, 8, 13, 14). Besides IL-6, SARS-CoV-2 infection leads to the secretion of high amounts of IL-1β, IL-2, IL-4, IL-7, IL-10, monocyte chemoattractant protein (MCP)-1, and tumor necrosis factor (TNF)-α into the plasma of COVID-19 patients (3, 15–17).

Thus far, early immune responses upon SARS-CoV-2 infection are not yet fully understood. Furthermore, little is known about how innate immune cells contribute to the massive cytokine production and pathogenesis in COVID-19. Feng et al. revealed that CD169^+^ macrophages within the spleen and lymph nodes are infected with SARS-CoV-2 and might contribute to viral replication and spread (18). Histological and single cell analyses of tissue samples identified CD169^+^ macrophages as a potent source of IL-6 in the lung and hilar lymph nodes (14, 18). Previous studies showed that alveolar macrophages and lung-resident dendritic cells (DC) provide the first line of defense against respiratory viral infections (19). A recent study demonstrated that DC from acute COVID-19 patients are functionally impaired in maturation and T cell activation indicating an attenuated adaptive T cell response (20).

The current study aims to uncover the role of human innate immune cells, DC and macrophages, upon SARS-CoV-2 infection. Identification of cells permissive to SARS-CoV-2 and analyses of the activation status and cytokine production of these cells will help to understand the pathogenesis upon early infection.

## Materials and Methods

### Cell Isolation and *In Vitro* Differentiation

Buffy coats obtained from the German Red Cross blood donation center (Deutsches Rotes Kreuz (DRK) Blutspendedienst) (Frankfurt am Main, Germany) were used for isolation of peripheral blood mononuclear cells (PBMCs) by Biocoll density gradient centrifugation. Briefly, a human buffy coat is diluted with PBS and carefully coated on a separation medium (Biocoll). After centrifugation, the blood components are separated according to their density. The PBMCs layer can be collected and used for further cell separation. Monocytes were purified from freshly isolated PBMCs using CD14 MicroBeads (Miltenyi Biotec, Bergisch Gladbach, Germany) and *in vitro* differentiated to obtain myeloid DC (mDC), type 1 (M1), or type 2 (M2) macrophages. Purified monocytes were cultured for 5 days in 24-well plates (1x10^6^cells/mL) using serum-free X-Vivo 15 medium (including L-Glutamine, gentamicin, and phenol red; Lonza, Verviers, Belgium) in the presence of 1,000 U/mL GM-CSF plus 1,000 U/mL IL-4 (both CellGenix, Freiburg, Germany) for the generation of mDC, in the presence of 10 ng/mL GM-CSF for the generation of M1 macrophages, or the presence of 30 ng/mL M-CSF (R&D Systems, Wiesbaden, Germany) for the generation of M2 macrophages.

### Virus and Stimuli

SARS-CoV-2 (isolate MUC IMB-1) and SARS CoV (isolate Frankfurt-1, 2002) were propagated on VeroE6 cells. Infections were carried out with either MOI 0.01 or 0.1 for 4 h, 24 h, or 48 h (indicated in the respective figure). All infection experiments were performed under biosafety level (BSL)-3 conditions at the Paul-Ehrlich-Institut.

For co-infection experiments, LPS (Salmonella abortus equi, Sigma, St. Louis, MO, USA; 0.1 µg/mL), Pam3CysSK4 (EMC microcollections; 1µg/mL), flagellin (kindly provided by Stefan Schülke; 10 µg/mL) (21), polyionosinic-polycytidylic acid (poly(I:C) HMW; InvivoGen, Toulouse, France; 1µg/mL), and R848 (InvivoGen; 10 µg/mL) were used. Vesicular stomatitis virus Indiana (VSV, Mudd-Summers isolate strain Indiana) was originally obtained from D. Kolakofsky and propagated on BHK-21 cells (ATCC^®^ CCL-10. Rift Valley Fever virus (RVFV) clone 13 and Sendai virus (SeV) were propagated on Vero cells (ATCC CCL-81); influenza A viruses (strain A/PR/08/34 (H1N1-1), strain A/HH/04/09 (H1N1-2), strain A/Udorn/307/72 (H3N2-1), and strain A/HK/01/68 (H3N2-2)) were propagated on MDCK cells (ATCC CCL-34). RVFV, SeV, and influenza A viruses were kindly provided by Georg Kochs.

Stimulation with LPS (0.1 µg/mL) plus IFN-*γ* (1 U/mL; PeproTech Inc., Rocky Hill, NJ, USA), heat-inactivated cells (65°C for 10 min), or untreated cells were used as controls.

### *In Vitro* Stimulation and Quantification of Cytokine Production

**Figure 1:**
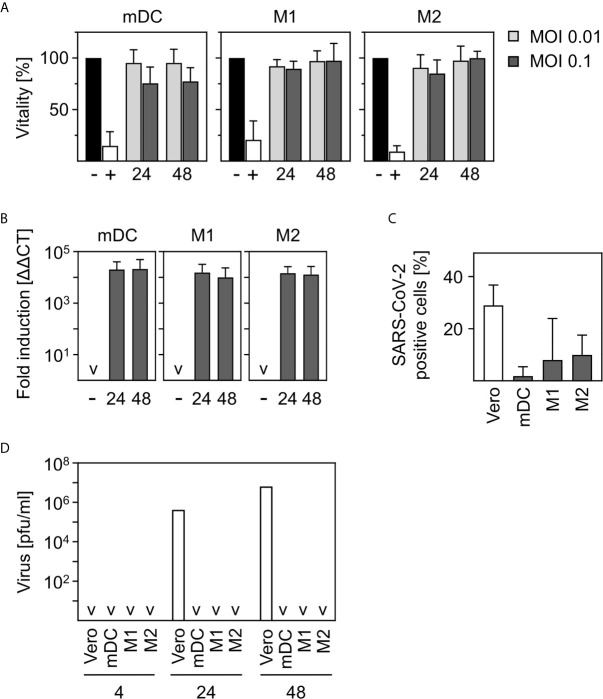
SARS-CoV-2-infected human innate immune cells are viable and show no productive infection. **(A)** *In vitro* differentiated mDC, M1-, or M2 were infected with multiplicity of infection (MOI) 0.01 or 0.1 of SARS-CoV-2 for 24 and 48 h. Analyses of cell viability were performed by flow cytometry by defining cells present in the live gate of the forward/sideward scatter (FSC/SSC) as viable cells (*n* = 4-12, performed in 1-3 independent experiments). Untreated cells (-) or heat-inactivated cells (+) served as controls. **(B)** mDC, M1-, or M2 macrophages were infected with SARS-CoV-2 (MOI 0.1) for 24 or 48 h. Following RNA extraction, q-PCR analyses were performed for SARS-CoV-2 N1 (*n* = 8-9, performed in 2 independent experiments; ˅ = not detectable). **(C)** In order to investigate if SARS-CoV-2 is capable of infecting mDC, M1-, and M2 macrophages, an intracellular staining using SARS nucleoprotein antibody was performed. SARS-CoV-2-stained untreated cells (negative control, not shown) and VeroE6 cells (positive control) served as controls. SARS-CoV-2 N protein positive cells are given in percentage positive cells (*n* = 3-4, in 1-2 experiments) **(D)** To determine if infection was productive mDC, M1-, and M2 macrophages were infected with SARS-CoV-2 (MOI 0.1) or were left untreated as negative control (not shown). Cells were washed twice 4 h post infection. SN were harvested 4, 24, or 48 hpi and analyzed for the presence of newly formed viral particles by plaque assay. SN of SARS-CoV-2 infected VeroE6 cells served as positive control. (*n* = 4, performed in 1 experiment; ˅ = not detectable). Error bars indicate standard deviations.

For infection, *in vitro* differentiated mDC, M1-, or M2 macrophages were seeded at 1x10^6^ cells/well in 24-well culture plates in 1 mL medium. SARS-CoV-2 infections were performed either with MOI 0.01 (**Figures 1** and **3**) or MOI 0.1 (all figures). After 24 or 48 hours post infection (hpi), cells were harvested for FACS analyses or q-PCR analyses. Cell-free supernatant (SN) was collected and analyzed using enzyme-linked immunosorbent assay (ELISA) kits allowing the determination of human IL-6, TNF-α (both from R&D), and IFN-α (PBL).

### Quantitative Real-Time PCR (qPCR)

Total RNA was prepared from infected cells using Trizol (Invitrogen)/chloroform extraction. Samples were treated with DNaseI (Roche) for 60 min at 37°C. Absence of genomic DNA contamination was confirmed by standard PCR using a glyceraldehyde 3-phosphate dehydrogenase (GAPDH)-specific primer pair. The primers amplify a fragment without crossing an intron and thus the presence of genomic DNA would be detectable by the amplification of a fragment with a size of 500 kb on an agarose gel. Target- and reference-mRNA levels were examined by q-PCR using QuantiFast SYBR Green PCR kit (Qiagen). Primer pairs used were the following: GAPDH forward 5’-ACCACAGTCCATGCCATCAC-3’, GAPDH reverse 5’-TCCACCACCCTGTTGCTGTA-3’, SARS-CoV-2 N1 protein forward 5’-GACCCCAAAATCAGCGAAAT -3’, SARS-CoV-2 N1 protein reverse 5’-TCTGGTTACTGCCAGTTGAATCTG-3’, IFN-α forward 5’- GACTCCATCTTGGCTGTGA-3’, IFN-α reverse 5’- TGATTTCTGCTCTGACAACCT-3-3’, TNF-α forward 5’-GGCTCCAGGCGGTGCTTGTTC-3’, TNF-α reverse 5’-AGACGGCGATGCGGCTGATG-3’, IL-6 forward 5’-ATTCGGTACATCCTCGAC-3’, IL-6 reverse 5’-GGGGTGGTTATTGCATC-3’. The expression levels of all target genes were normalized against GAPDH (ΔCt). Gene expression values were calculated based on the ΔΔCt method, using the mean of the untreated control group as calibrator to which all other samples were compared. Relative quantities (RQ) (22) were determined using the equation RQ=2^-ΔΔCt^.

### Plaque Assay

To determine if SARS-CoV-2 infection is productive in mDC, M1-, and M2 macrophages, cells were infected for 4 h and then washed twice to ensure that no remaining viral particles were present in the freshly added medium. SN were collected after 24 h or 48 h. To determine the viral load within the SN, plaque assay analyses were performed using VeroE6 cells (ATCC CRL-1586) as described in (23, 24). In brief, serial dilutions of SN were prepared, incubated on 2x10^5^ Vero E6 cells for 3 days, and subsequently stained with crystal violet. Number of plaques formed were used to calculate plaque forming units (PFU) per mL.

### Flow Cytometry

mDC, M1-, and M2 macrophages were collected by up and down pipetting followed by using cell scrapers. Flow cytometric analyses were performed using mouse anti-human CD11b-FITC (clone M1/70.15.11.5, Miltenyi Biotec, Bergisch Gladbach, Germany (working dilution 1 µL), anti-human CD11c-APC (clone B-ly6, BD Bioscience, Heidelberg, Germany(working dilution 1 µL)), anti-human CD14-PacBlue (clone M5E2, BD Bioscience, Heidelberg, Germany (working dilution 1 µL)), anti-human CD40-PacBlue (clone 5C3, Biolegend, Fell, Germany (working dilution 1 µL)), anti-human CD64-FITC (clone 10.1, BD Pharmingen, San Jose, USA (working dilution 1 µL)), anti-human CD68-PE (clone Y1/82A, BioLegend, Fell, Germany), anti-human CD80-APC (clone MEM-233, EuroBioSciences, Friesoythe, Germany (working dilution 1 µL)), anti-human CD83-FITC (clone BH15e, BD Bioscience, Heidelberg, Germany (working dilution 2 µL), anti-human CD86-PE (clone IT2.2, Biolegend, Fell, Germany (working dilution 1 µl (1:10))), anti-human CD169-APC (clone 7-239, Biolegend, Fell, Germany (working dilution 1 µL)), anti-human CD206-Brilliant Violet 711 (clone 15-2, Biolegend, San Diego, USA (working dilution 1 µL)), and anti-human HLA-DR-APCCy7 (clone L243, Biolegend, Fell, Germany (working dilution 1 µL (1:4))) antibodies. Prior to intracellular staining with SARS nucleocapsid protein antibody (Rockland, USA; (working dilution 1 µL (1:10))), cells were fixed and permeabilized using BD Cytofix/Cytoperm™ according to manufacturer’s instructions. Chicken anti-rabbit IgG(H+L) cross-adsorbed AlexaFLuor488 (working dilution 1µl (1:2)) was used as secondary antibody for detection. FACS analyses were performed on LSRFortessa and LSR II flow cytometers. Data were analyzed by BD FACSDiva software version 8.0.1 (BD Biosciences, Heidelberg, Germany) and FlowJo software version 7.6.5, or 10.0.8 (Tree Star, Ashland, OR, USA).

### Statistics

All statistical analyses were performed with GraphPad Prism 8.1.2. We analyzed our raw data using the D’Agostino & Pearson test for normal distribution. All experimental data passed the normality test and were found to have a normal distribution. For statistical analyses, the Wilcoxon signed-rank test (when data were normalized to the negative control) or paired t-test (all other data) were used. Values with p ≤ 0.05 are statistically significant which is illustrated by the number of stars: (*) for p ≤ 0.05, (**) for p ≤ 0.01, (***) for p ≤ 0.001, and (****) for p ≤ 0.0001.

## Results

### Human Innate Immune Cells Are Viable and Not Productively Infected by SARS-CoV-2

The early immune response upon infection with SARS-CoV-2 and the contribution of innate immune cells to the formation of the observed cytokine storm in COVID-19 patients is not yet fully understood. Hence, we aim to uncover the role of different subtypes of human innate immune cells, DC and macrophages, upon infection with SARS-CoV-2.

In order to investigate the viability of innate immune cells upon infection with SARS-CoV-2, we infected *in vitro* differentiated mDC as well as M1-, and M2 macrophages using a multiplicity of infection (MOI) of 0.01 and 0.1 for the indicated time points. Viability of untreated cells was set to 100% and heat-treated (65°C for 10 min) cells served as control. As shown in **Figure 1A**, SARS-CoV-2 infection did not alter viability of mDC, M1-, or M2 macrophages after infection for 24 h and 48 h. To analyze susceptibility, mDC, M1-, and M2 macrophages were infected with SARS-CoV-2 (MOI 0.1) for 24 h and 48 h. Cells were analyzed for the presence of SARS-CoV-2 N1 RNA by q-PCR. Results given in **Figure 1B** show a 10,000-fold upregulation of viral RNA expression upon infection of mDC, M1-, and M2 macrophages. However, no increase of viral RNA over time could be detected. To analyze if SARS-CoV-2 is able to infect mDC, M1-, and M2 macrophages, intracellular stainings using an anti-SARS nucleocapsid (N) protein antibody were performed. VeroE6 cells were used as control. As given in **Figure 1C**, no SARS-CoV-2 N protein expressing mDC could be detected. In contrast, M1 macrophages showed a slight upregulation which was even more pronounced for M2 macrophages. Of note, although 7.9% (M1) and 9.9% (M2) of cells were positive, SARS-CoV-2 N protein expression was not observed for all donors tested (see **Supplementary Figure 1**). Finally, to clarify if the cells could be productively infected, plaque assay analyses using supernatants (SN) of SARS-CoV-2-infected mDC, M1-, and M2 macrophages were performed. For this, cells were infected with MOI 0.1 for 4 h to enable infection and then washed twice before further incubation. To ensure that no remaining viral particles were present after washing, SN of 4 h infected cells were collected and included in the measurement. Highly permissive VeroE6 cells served as positive control. Interestingly, no newly formed viral particles were detected within the SN of mDC, M1-, or M2 macrophages 24 or 48 h post infection (**Figure 1D**) indicating that the cells are infected without producing progeny. In contrast, VeroE6 cells showed robust formation of newly synthesized viral particles. In conclusion, innate immune cells are susceptible to SARS-CoV-2 infection, but the infection is not productive.

### M2 Macrophages Show a Phenotype Comparable to CD169^+^ Alveolar Macrophages

For SARS-CoV-2, Feng et al. showed that CD169^+^ macrophages infiltrate the lungs of COVID-19 patients and are positive for SARS-CoV-2 N protein, indicating a possible contribution of these cells to a SARS-CoV-2 related cytokine production. In addition, it was shown that macrophages within the spleen and lymph nodes of SARS-CoV-2 infected patients were positive for CD68 (18).

**Figure 2:**
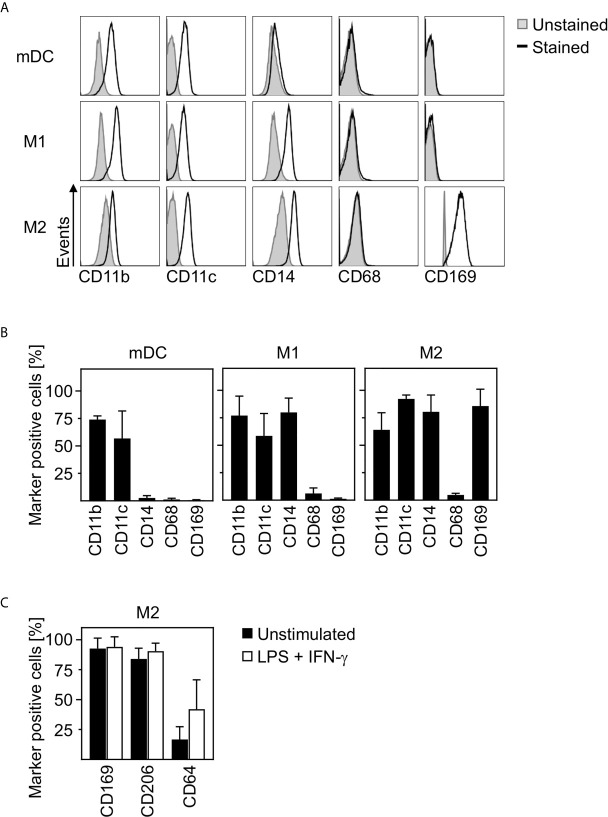
Characterization of human innate immune cells. *In vitro* differentiated mDC, M1-, and M2 macrophages were analyzed for surface expression of CD11b, CD11c, CD14, CD68, and CD169 by flow cytometry. Unstained cells were used as negative control. **(A)** One representative donor for each cell type is shown (grey filled = unstained; black line = stained). **(B)** Graphs show percentages of marker positive mDC, M1-, and M2 macrophages (*n* = 4-10, performed in 2-4 independent experiments). **(C)** In order to further investigate the phenotype of *in vitro* differentiated M2 macrophages, cells were analyzed for CD169, CD206, and CD64 expression by flow cytometry. Cells were either left untreated (black bars) or treated with LPS/IFN-*γ* (white bars) as controls (*n* = 6, performed in 2 independent experiments). Error bars indicate standard deviations.

In order to clarify how closely related *in vitro* differentiated innate immune cells are to cells infiltrating the lungs, spleens, and lymph nodes of SARS-CoV-2 infected patients, we characterized the phenotype of *in vitro* differentiated mDC, M1-, and M2 macrophages. For this, we analyzed the expression of surface markers CD11b, CD11c, CD14, CD68, and CD169 by flow cytometry. Histograms of a representative donor (**Figure 2A**) and a summary of the surface marker expression profile for each cell type (**Figure 2B**) are shown. Analyses demonstrated that mDC, M1- and M2 macrophages did not express CD68. In addition, neither CD11b^+^CD11c^+^ mDC nor CD11b^+^CD11c^+^CD14^+^ M1 macrophages expressed CD169. Interestingly, anti-inflammatory CD11b^+^CD11c^+^CD14^+^ M2 macrophages were positive for CD169. Mitsi et al. revealed that human alveolar macrophages express combined classical M1 and M2 surface markers. Here, the majority of alveolar macrophages were CD206^hi^ and expressed CD64. Furthermore, it was shown that tissue resident alveolar macrophages express markers such as CD11c, CD64, and CD169 (25, 26). In order to investigate how comparable M2 macrophages are to alveolar macrophages, a further in depth characterization of *in vitro* differentiated CD169^+^ M2 macrophages was performed. Flow cytometric analyses using CD169, CD64, and CD206 revealed that >80% were positive for CD169 and CD206, while ~16% expressed CD64. Of note, CD64 expression was further increased up to ~42% (**Figure 2C**) upon treatment with LPS/IFN-*γ*.

In conclusion, these data strongly indicate that *in vitro* differentiated anti-inflammatory M2 macrophages show a phenotype comparable to CD169^+^CD206^+^ alveolar lung macrophages.

### M2 Macrophages Upregulate CD40 and Downregulate CD86 and MHC-II Expression Upon SARS-CoV-2 Infection

**Figure 3:**
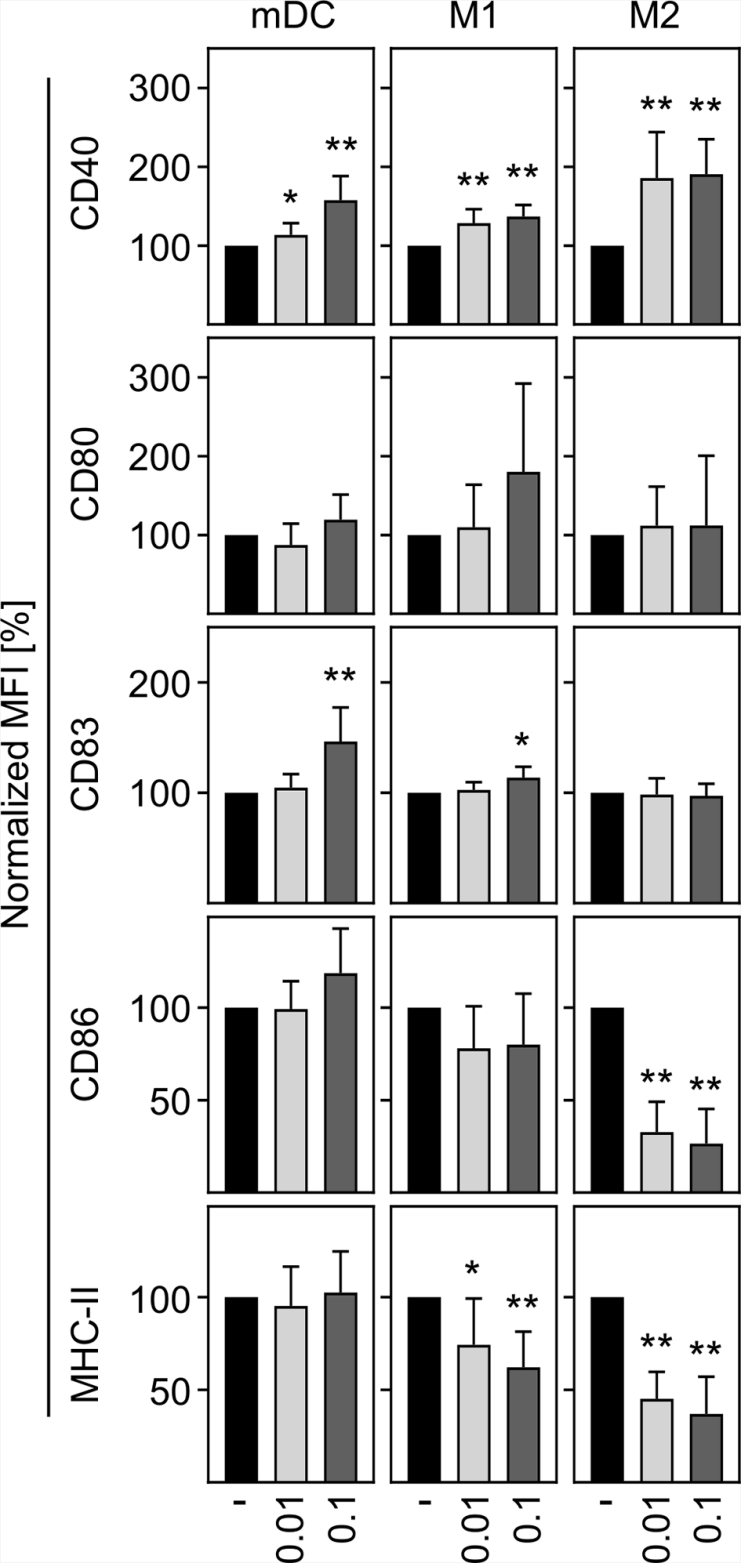
Activation status of mDC, M1-, and M2 macrophages upon infection with SARS-CoV-2. To analyze the activation status of mDC, M1-, and M2 macrophages upon SARS-CoV-2 infection, cells were infected with either MOI 0.01 or MOI 0.1 for 24 h, stained for the activation/maturation markers CD40, CD80, CD83, CD86, and MHC-II, and analyzed by flow cytometry. Bar graphs show MFI of the respective marker. Untreated cells were normalized to 100 (black bars = untreated; light grey bars = MOI 0.01; dark grey bars = MOI 0.1; *n* = 8, performed in 2 independent experiments). Error bars indicate standard deviations. * ≤ 0.05; ** ≤ 0.01 (Wilcoxon signed-rank test).

In a next step, we aimed to investigate the activation status of *in vitro* differentiated human innate immune cells upon SARS-CoV-2 infection. To ensure that cells can be activated, mDC, M1-, and M2 macrophages were treated with LPS plus IFN-*γ* as positive control (LPS/IFN-*γ*) and analyzed for CD40 expression. Untreated cells served as negative control. Histograms of a representative donor are shown in **Supplementary Figure 2A**. Analyses of the mean fluorescence intensity (MFI) revealed a significant upregulation of CD40 expression by mDC, M1-, and M2 cells upon treatment, indicating that all immune cells were responsive (**Supplementary Figure 2B**). To uncover the activation status of mDC, M1-, and M2 macrophages upon SARS-CoV-2 infection, cells were infected with an MOI 0.01 or MOI 0.1 for 24 h and analyzed for the expression of the activation or maturation markers CD40, CD80, CD83, CD86, and MHC-II. As given in **Figure 3**, mDC and M1 macrophages showed a statistically significant but rather minor upregulation of CD40 at both MOIs tested. This SARS-CoV-2 induced upregulation of CD40 was more pronounced upon infection of M2 macrophages. Expression of CD83 was slightly upregulated on mDC and M1 macrophages upon infection with SARS-CoV-2 (MOI 0.1). Furthermore, SARS-CoV-2 infection slightly decreased the expression of MHC-II on M1 macrophages. Of note, SARS-CoV-2 infected M2 macrophages demonstrated a statistically significant decreased expression of the activation markers CD86 and MHC-II at both MOIs tested (both p = 0.0078; **). Thus, SARS-CoV-2 infection slightly upregulates CD40 expression on innate immune cells and decreases CD86 and MHC-II expression on M2 macrophages.

### *In Vitro* Differentiated Innate Immune Cells Are Not Contributing to Cytokine Production Upon SARS-CoV-2 Infection

Studies using SARS-CoV spike protein demonstrated that murine macrophages produce IL−6 and TNF-α *in vitro* (27). Furthermore, severe infections with SARS-CoV-2 are often associated with a cytokine storm resulting in high levels of IL-6 and TNF-α in the patients (3, 15–17). The source of this massive induction of pro-inflammatory cytokines has not yet been elucidated.

**Figure 4:**
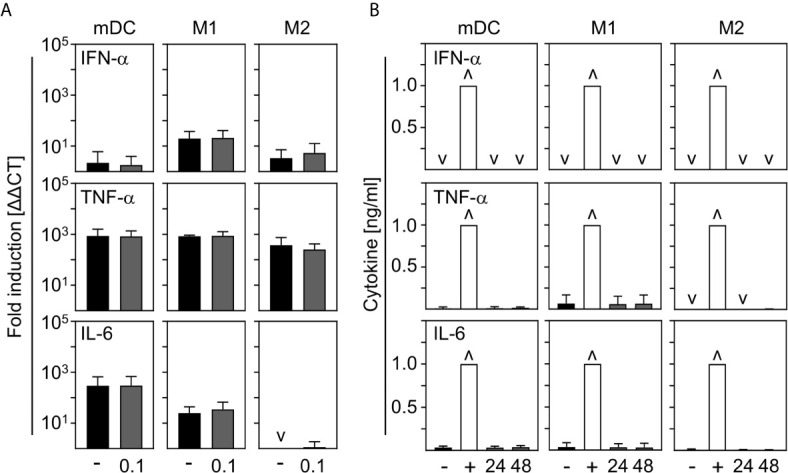
Cytokine expression by mDC, M1-, and M2 macrophages upon infection with SARS-CoV-2. **(A)** SARS-CoV-2 infected mDC, M1-, and M2 macrophages (all with MOI 0.1) were harvested 24 hpi and induction of IFN-α, TNF-α; and IL-6 was analyzed by q-PCR. Untreated cells served as control (*n* = 4-5, performed in 1 experiment; ˅ = not detectable). **(B)** SN of SARS-CoV-2 infected mDC, M1-, and M2 macrophages were harvested 24 or 48 hpi. Untreated cells (-) and RVFV clone 13-infected cells (for IFN-α; +), or LPS/IFN−*γ*−treated cells (for TNF-α and IL-6; +) served as controls. Subsequent analysis of the presence of IFN-α, TNF-α, and IL-6 were performed by an ELISA method according to the manufacturers’ instructions (*n* = 4-9, performed in 1-2 independent experiments; ˅ = not detectable; ˄ = saturation). Error bars indicate standard deviations.

In order to investigate the contribution of innate immune cells to the production of the most prominent cytokines expressed during COVID-19, we analyzed the production of pro-inflammatory IFN-α, TNF-α, and IL-6 by *in vitro* differentiated innate immune cells upon SARS-CoV-2 infection. For this, mDC, M1-, and M2 macrophages were infected with SARS-CoV-2 (MOI 0.1) for 24 h and analyzed by q-PCR. Untreated cells served as control. As shown in **Figure 4A**, no IFN−α, TNF-α, or IL-6 mRNA was induced upon infection. In a next step, cells were infected with SARS-CoV-2 (MOI 0.1) for 24 h and 48 h and cytokines in the SN were analyzed by ELISA. Infections with Rift Valley Fever virus (RVFV) clone 13 (for IFN-α) or stimulation with LPS/IFN-*γ* (for TNF-α and IL-6) were performed as positive controls. In correlation with q-PCR data, no expression of IFN−α; TNF-α, and IL-6 could be observed upon infection of mDC, M1-, and M2 macrophages with SARS-CoV-2 (**Figure 4B**). Surprisingly, these results show that neither mDC nor M1- nor CD169^+^CD206^+^ M2 macrophages are stimulated to early cytokine production upon SARS-CoV-2 infection *in vitro*.

### Co-Infection With SARS-CoV-2 Induces Only a Minor Increase in IL-6 Production

**Figure 5:**
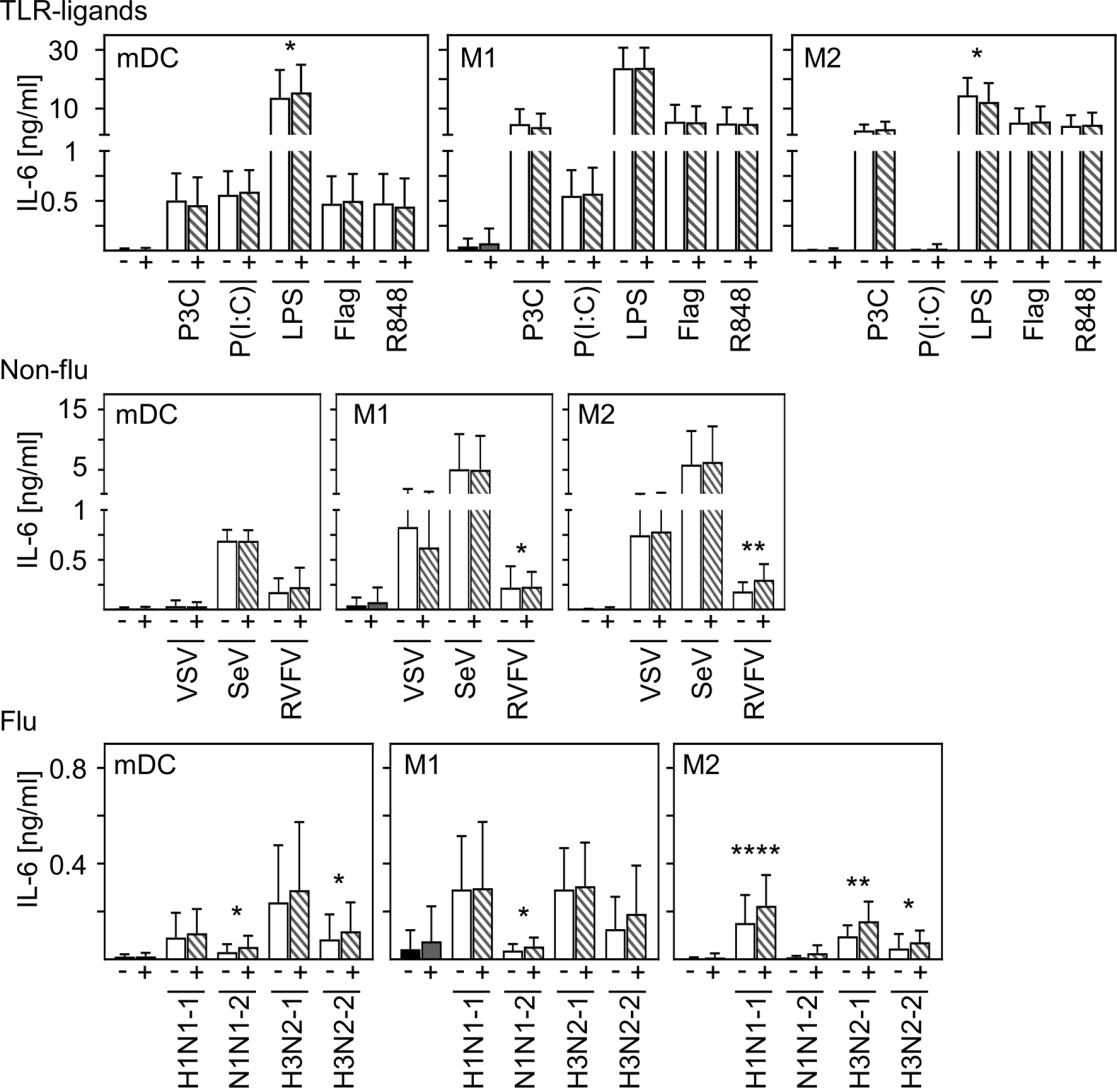
Co-infection with SARS-CoV-2 does not substantially enhance virus-induced IL-6 production of M2 macrophages. *In vitro* differentiated mDC, M1-, and M2 macrophages were either treated with the TLR ligands Pam3Cys (TLR2), p(I:C) (TLR3), LPS (TLR4), flagellin (TLR5), or R848 (TLR7/8 [upper row]; the non-flu viruses VSV, SeV, or RVFV (all in MOI 0.1; middle row); or the flu viruses H1N1-1; H1N1-2, H3N2-1, or H3N2-2 (all in MOI 0.1; lower row) alone (white bars), or co-infected with SARS-CoV-2 (MOI 0.1) (striped bars). Of note, infection with SARS-CoV-2 was performed 4 h prior to treatment. SN were collected 24 h post infection and analyzed for the presence of IL-6 by ELISA. (*n* = 9-16, performed in 2-3 independent experiments). Error bars indicate standard deviations. * ≤ 0.05; ** ≤ 0.01; **** ≤ 0.0001 (paired t-test).

In contrast to other studies (28, 29), we could not detect production of pro-inflammatory IL-6 upon *in vitro* infection of mDC and macrophages. To examine to what extent SARS-CoV-2 modulates cytokine production induced by other stimuli or infections, co-infection experiments were carried out. For this, *in vitro* differentiated mDC, M1-, or M2 macrophages were pre-infected with SARS-CoV-2 (MOI 0.1) for 4 h and subsequently treated with the indicated stimuli for another 20 h. For these experiments, several Toll-like receptor (TLR) ligands and different ssRNA encoded viruses (non-flu and flu viruses) were tested. SN were harvested 24 hpi and analyzed for the presence of IL-6 by ELISA. As given in **Figure 5**, infection with SARS-CoV-2 influenced neither the effect of most TLR ligands- (upper row) nor VSV- nor Sendai Virus (SeV)-induced (middle row) IL-6 production by all cell types tested. Furthermore, SARS-CoV-2 induced only a slight increase of LPS-mediated IL-6 production by mDC and RVFV-mediated IL-6 secretion by M1 and M2 macrophages. Using different subtypes of flu-viruses (H1N1 and H3N2; two strains for each, lower row) revealed that co-infection with SARS-CoV-2 also induced just a slight increase in IL-6 production by all cell types tested. Interestingly, this increase in IL-6 production was most pronounced upon co-infection of M2 macrophages. Comparison of the individual donors regarding IL-6 production revealed that this trend could be observed upon infection of M2 macrophages with RVFV, H1N1−1, and H3N2-1 for each donor tested (**Supplementary Figure 3**). In line with this, SARS-CoV-2 slightly increased the expression of IFN-α by H1N1-infected M2 macrophages (data not shown).

In conclusion, these data indicate that infection of human innate immune cells with SARS-CoV-2 mediates only a slight increase in the release of pro-inflammatory IL-6 induced by secondary infections or stimulation.

## Discussion

Severe infections with SARS-CoV-2 are often accompanied by a massive cytokine storm with IL-6 as the driver of inflammation present in the lungs and serum of COVID-19 patients (3, 8, 13, 14). The role of innate immune cells such as DC and macrophages upon infection with SARS-CoV-2 and their contribution to the production of pro-inflammatory cytokines is still under debate. Post-mortem analyses of COVID-19 patients revealed an infiltration of CD169^+^ macrophages expressing SARS-CoV-2 N protein within the lung (18). In line with this, analysis of bronchoalveolar lavage fluid from patients with severe COVID-19 revealed a high proportion of macrophages compared to healthy donors (30). However, other studies could not detect an excessive infiltration of neutrophils and macrophages into the lungs of COVID-19 patients (31). Single cell RNA sequencing of pulmonary tissue from COVID-19 patients revealed an expansion of interstitial and monocyte-derived macrophages but not alveolar macrophages. Therefore, it is not yet clear if circulating monocytes and macrophages are targets for SARS-CoV-2 infection and a potential source for pro-inflammatory cytokines (28).

SARS-CoV-2 was shown to infect and replicate in human lung tissue, mainly type I and type II pneumocytes, but also in alveolar macrophages (24, 32). Interestingly, the diverse expression of ACE2 in macrophages among individual donors was assumed to drive severity of a SARS-CoV-2 infection (33). Recent studies showed that, besides ACE2, other receptors, such as CD147 or CD26, might facilitate entry of SARS-CoV-2 into the host cell (9–11). These receptors are also present on innate immune cells such as DC or macrophages and therefore might promote infection. Our results demonstrated that *in vitro* differentiated mDC, M1-, and M2 macrophages are infected by SARS-CoV-2 but do not form newly synthesized viral particles (see **Figure 1**). This was also shown by other studies revealing DC and macrophages to be susceptible to SARS-CoV-2 infection without productive virus replication or cytopathic effect (28, 29, 34, 35). In addition to this, human primary blood mononuclear cells and THP-1 cells did not support productive SARS-CoV-2 replication (36). Of note, alveolar macrophages are known to be not productively infected by most seasonal influenza strains (37).

Analysis of the activation status upon SARS-CoV-2 infection showed an upregulation of CD40 on mDC, M1-, and M2 macrophages (**Figure 3**). In correlation with this, a greater proportion of activated macrophages and monocytes were detected in COVID-19 patients (30). CD40 expression in COVID-19 patients was shown to be restricted to myeloid cells while other cells seemed to have downregulated CD40 (38). In contrast, a study performed by Sachez Cerillo et al. did not find any changes in CD40 expression on CD1c^+^ DC and CD141^+^ DC (38). Furthermore, our results demonstrate that despite an upregulation of CD40, CD169^+^ M2 macrophages downregulate CD86 and MHC-II expression upon infection with SARS-CoV-2 (**Figure 3**). This correlates with a recent study showing a significantly lower HLA-DR and CD86 expression on DC in SARS-CoV-2 infected patients compared to healthy donors (20). In addition, monocytes revealed a remarkably reduced HLA-DR expression in hospitalized patients (28, 39).

Whereas cell viability showed no clear difference upon SARS-CoV and SARS-CoV-2 infection (**Supplementary Figure 4A**), SARS-CoV did not alter CD40 expression in all cell types tested (**Supplementary Figure 4B**). Furthermore, reductions of CD86 and MHC-II in CD169^+^ M2 macrophages were observed to a lesser extent upon SARS-CoV infection. Thus, our data indicate that the impact of SARS-CoV infection on the activation status of innate immune cells was less pronounced when compared to SARS-CoV-2.

Recent studies showed that the observed cytokine storm in COVID-19 patients might be an important factor in disease progression. The role of innate immune cells in the production of pro-inflammatory cytokines is still under discussion. Surprisingly, we could not observe production of pro-inflammatory cytokines by SARS-CoV-2 infected mDC, M1-, or M2 macrophages *in vitro* (see **Figure 4**). In stark contrast to our data, several *ex vivo* and *in vitro* studies showed that SARS-CoV-2 infected macrophages, but not DC, are capable of producing pro-inflammatory cytokines such as IL-6 or TNF-α (18, 28–31). Moreover, a recent study performed by Zheng et al. demonstrated *in vitro* differentiated mDC and macrophages to produce IFN-α, TNF-α, and IL-6 upon SARS-CoV-2 infection (35).In correlation with this, high levels of IL-6 in critically ill COVID-19 patients are assumed to be produced by highly inflammatory macrophages (40). It was previously shown that SARS-CoV-2 efficiently suppresses the antiviral type I IFN response in monocytes and macrophages which might result in a delay of viral clearance and contribute to COVID-19 pathogenesis (29, 30, 41). In line with this, alveolar macrophages did not produce type I IFN upon *in vitro* infection with SARS-CoV-2 (42). Furthermore, Delagado-Ortega et al. revealed that rather epithelial cells than alveolar macrophages are capable to produce cytokines such as IFN-β upon swine influenza infection (43). Of note, in most severe cases of COVID-19, the immune dysregulation leading to the massive cytokine release is observed around day 7-10 post infection supporting our data that cells others than innate immune cells are the source of the first wave of pro-inflammatory cytokines (17, 44, 45).

Co-infections can cause altered disease severity and transmissibility by viral interference or modulation of viral replication or cytokine production (46). To investigate the influence of SARS-CoV-2 infection on secondary infections, co-infection/-stimulation experiments were performed. Our data revealed that SARS-CoV-2 infection did not significantly increase IL-6 production induced by most stimuli tested. Flu-induced IL-6 production was slightly enhanced in M2 macrophages. This minor increase was statistically significant but whether the levels of induced IL-6 are of biological relevance is not clear yet. A study by Ma et al. demonstrated a more severe inflammatory response and organ injury in critically ill COVID-19 patients when co-infected with influenza A virus. The authors claim that co-infection may lead to an earlier and stronger cytokine storm. In correlation with our data, no differences in IL-6, TNF-α, and other clinical parameters, such as white blood cell counts, levels of c-reactive protein (CRP), alanine aminotransferase (ALT), aspartate transaminase (AST), lactate dehydrogenase (LDH), and creatinine, could be observed (47). *In vitro* and *in vivo* studies revealed that pre-infection with influenza strongly enhances infectivity of SARS-CoV-2 by boosting viral entry in the cells and by elevating viral loads resulting in a more severe lung damage in infected mice (48). In line with this, Stowe et al. reported that co-infection with influenza and SARS-CoV-2 was associated with an increased risk of death or severe disease (49). Furthermore, Saade et al. demonstrated that porcine reproductive and respiratory syndrome virus (PRRSV) does not infect respiratory epithelial cells but is able to inhibit replication of swine influenza A upon co-infection. In addition, type I IFN responses were modulated indicating that interaction between swine influenza A and its targets cells might are altered upon co-infection (50). In contrast, SeV, human rhinovirus (HRV3), human parainfluenza virus (HPIV), human respiratory syncytial virus (HRSV), and human enterovirus 71 (EV71) were all unable to facilitate SARS-CoV-2 infection of transgenic hACE2 mice (48). Interestingly, a large-scale assessment of SARS-CoV-2 co-infection with 38 other respiratory pathogens revealed that infection rates were significantly higher when patients were infected with SARS-CoV-2 when compared to SARS-CoV-2 negative patients (51).

In conclusion, our study demonstrated that *in vitro* differentiated human innate immune cells are able to be infected by SARS-CoV-2 without generating progeny. The activation status of all cell types analyzed changes only slightly upon SARS-CoV-2 infection. In addition, neither mDC nor M1- nor M2 macrophages were stimulated to produce pro-inflammatory cytokines upon SARS-CoV-2 infection. In contrast to several studies defining DC and macrophages as a potential source of IL-6 (18, 28–31), our data indicate that innate immune cells are unlikely the main source of the initial wave of pro-inflammatory cytokines upon severe SARS-CoV-2 infection. Even upon co-infection/-stimulation with a broad range of other TLR stimuli or viruses, production of pro-inflammatory cytokines by the cells analyzed was not substantially altered upon infection with SARS-CoV-2.

## Data Availability Statement

The original contributions presented in the study are included in the article/**Supplementary Material**. Further inquiries can be directed to the corresponding author.

## Author Contributions

MN designed experiments, performed experiments, and analyzed data. PG performed experiments and analyzed data. SK performed experiments. SOI performed experiments. GK discussed data and provided virus. ZW designed experiments and wrote the paper. MA designed experiments, performed experiments, analyzed data, and wrote the paper. All authors contributed to the article and approved the submitted version.

## Funding

This work was supported by the Bundesministerium fuer Bildung und Forschung (BMBF) through the Deutsches Zentrum für Luft- und Raumfahrt (DLR, grant number 01KI2077) to GK. Grant from the Federal State of Baden-Württemberg, Germany, to GK.

## Conflict of Interest

The authors declare that the research was conducted in the absence of any commercial or financial relationships that could be construed as a potential conflict of interest.

## Supplementary Material

Supplementary Figure 1 SARS-CoV-2 N protein expression is donor-dependent for M1 and M2 macrophages. mDC, M1-, and M2 macrophages were infected with SARS-CoV-2 (MOI 0.1) for 24 h and then stained intracellularly using an antibody directed against SARS-CoV-2 nucleoprotein. **(A)** Histogram overlays for each individual donor (D) are given with untreated cells (grey filled) and SARS-CoV-2 infected cells (black line). **(B)** VeroE6 cells served as control and were infected with SARS-CoV-2 (MOI 0.1) 24 h prior to intracellular staining.

Supplementary Figure 2 mDCs, M1 and M2 macrophages upregulate CD40 upon LPS/IFN-*γ* treatment. **(A, B)** To ensure that mDC, M1-, or M2 macrophages were capable of being activated and fully functional, cells were either left untreated or treated with LPS/IFN−*γ* (0.1 µg/mL and 1 U/mL, respectively) for 24 h. Cells were stained with an anti-CD40 antibody and analyzed by flow cytometry. **(A)** FSC/SSC dot plots and histograms showing CD40 expression of one representative donor are shown (black filled = untreated; dotted line = LPS/IFN-*γ* treated). **(B)** Graphs demonstrating the mean fluorescence intensity (MFI) of CD40 expression are given. Untreated cells were normalized to 100 (black bars = untreated; white bars = LPS/IFN-*γ* treated; *n* = 8; performed in 2 independent experiments). Error bars indicate standard deviations. ** ≤ 0.01 (Wilcoxon signed-rank test).

Supplementary Figure 3 Co-infection of SARS-CoV-2 synergistically increases virus-induced IL-6 production by M2 macrophages. Selected stimuli from **Figure 5** are given in a before and after plot showing the IL-6 expression of M2 macrophages from each individual donor without and with SARS-CoV-2 co-infection (*n* = 9-12, performed in 2 experiment). Error bars indicate standard deviations. ** ≤ 0.01 (paired t-test).

Supplementary Figure 4 Comparison of SARS-CoV-2 and SARS-CoV infected innate immune cells. **(A)** *In vitro* differentiated mDC, M1-, or M2 macrophages were infected with SARS-CoV (either MOI 0.01 or 0.1) and analyzed for viability by flow cytometry as described in **Figure 1**. SARS-CoV-2 infected cells from **Figure 1** are shown as comparison. Untreated cells (-) served as control. All viability data were normalized to untreated cells (*n* = 4-12; performed in 1-4 independent experiments). **(B)** mDC, M1-, and M2 macrophages were infected with SARS-CoV (MOI 0.1) for 24 h, stained for the activation/maturation markers CD40, CD80, CD83, CD86, and MHC-II, and analyzed by flow cytometry. Data for SARS-CoV-2 from **Figure 3** are shown as comparison. Untreated cells (-) served as control. Bar graphs show MFI of the respective marker normalized to untreated cells (*n* = 3-8; performed in 1-2 independent experiments). Error bars indicate standard deviations. * ≤ 0.05; ** ≤ 0.01 (Wilcoxon signed-rank test).

## Abbreviations

ACE2, Angiotensin-converting enzyme 2; AT2, type II alveolar epithelial cells; COVID-19, corona virus-induced disease; DC, dendritic cells; DRK, Deutsches Rotes Kreuz; ELISA, enzyme-linked immunosorbent assay; flu, influenza A; h, hours; hpi, hours post infection; IFN-α, interferon alpha; IL, interleukin; MCP-, monocyte chemoattractant protein 1; MERS, Middle East Respiratory Syndrome; MOI, multiplicity of infection; PRRSV, Porcine reproductive and respiratory syndrome virus; RVFV, Rift Valley Fever virus; SARS-CoV-2, severe acute respiratory syndrome coronavirus 2; SeV, Sendai virus; SN, supernatants; TLR, Toll-like receptor; TNF-α; tumor necrosis factor alpha; VSV, Vesicular stomatitis virus Indiana.
