# Supplementary figures and images for: Macrophages and Dendritic Cells Are Not the Major Source of Pro-Inflammatory Cytokines Upon SARS-CoV-2 Infection
Source: Front Immunol. 2021 May 26;12:647824. doi: 10.3389/fimmu.2021.647824 (PMC8187925; doi:10.3389/fimmu.2021.647824)

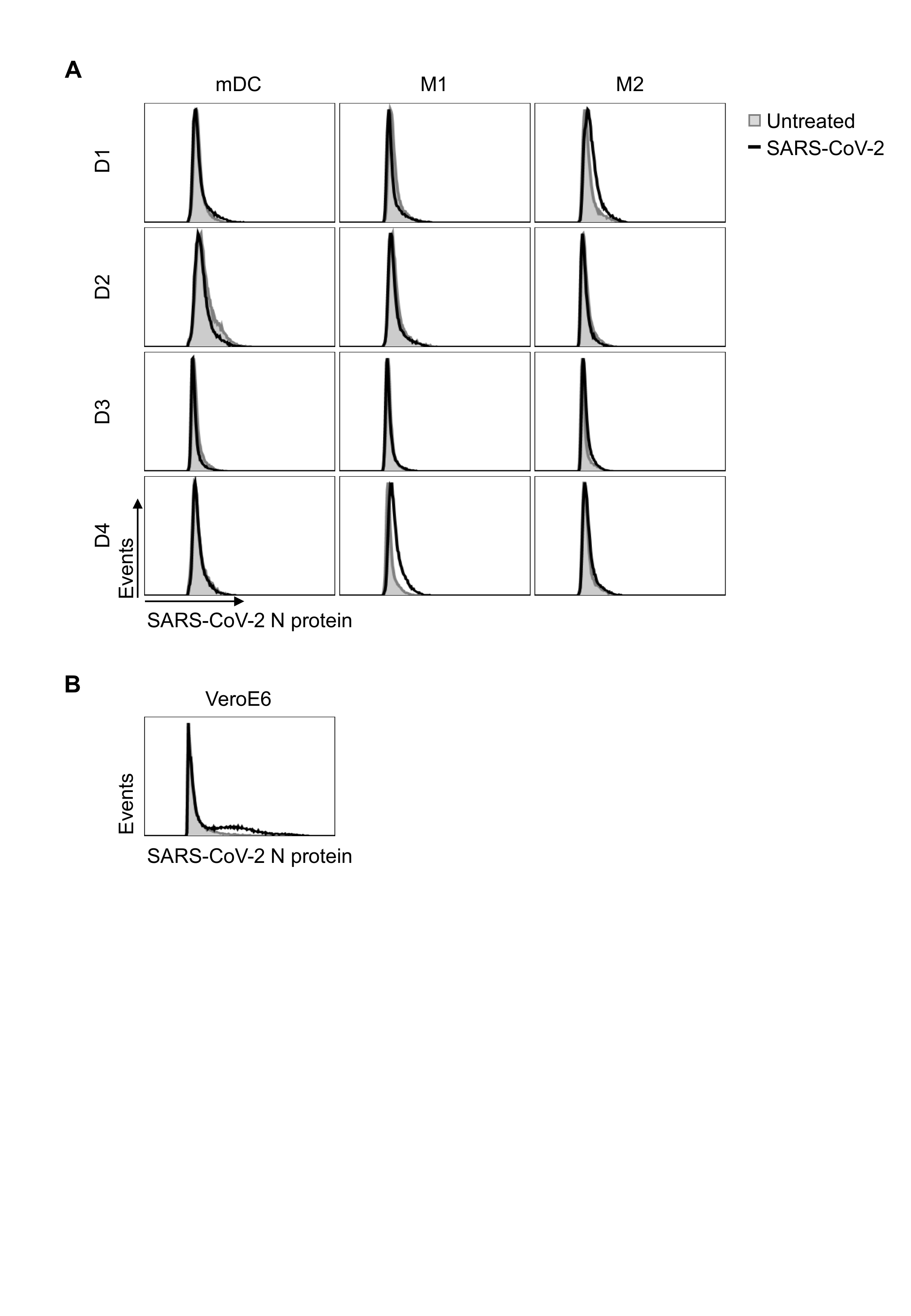

Supplement: Supplementary Figure 1 — SARS-CoV-2 N protein expression is donor-dependent for M1 and M2 macrophages. mDC, M1-, and M2 macrophages were infected with SARS-CoV-2 (MOI 0.1) for 24 h and then stained intracellularly using an antibody directed against SARS-CoV-2 nucleoprotein. (A) Histogram overlays for each individual donor (D) are given with untreated cells (grey filled) and SARS-CoV-2 infected cells (black line). (B) VeroE6 cells served as control and were infected with SARS-CoV-2 (MOI 0.1) 24 h prior to intracellular staining. [file Image_1.tif]

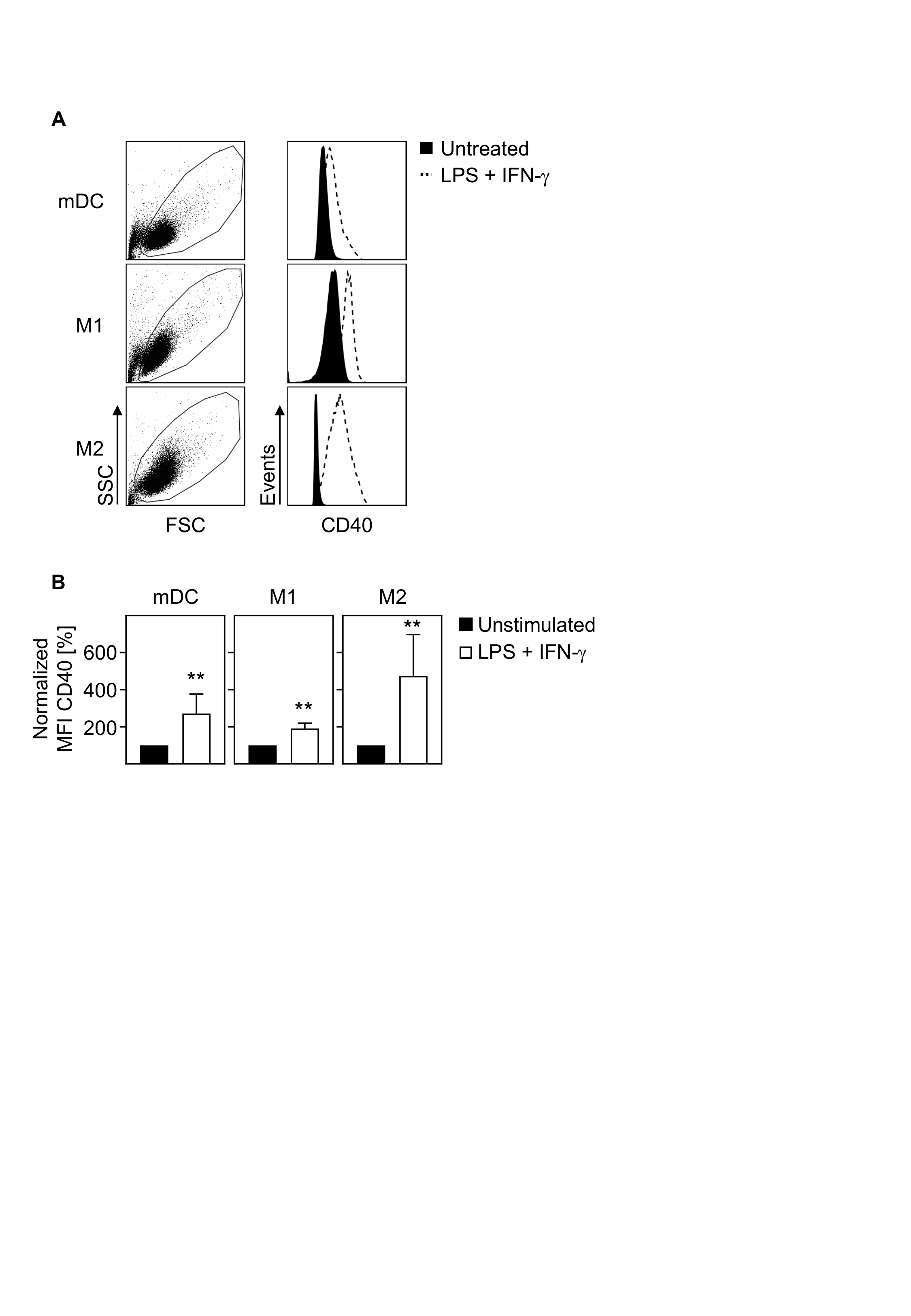

Supplement: Supplementary Figure 2 — mDCs, M1 and M2 macrophages upregulate CD40 upon LPS/IFN-γ treatment. (A, B) To ensure that mDC, M1-, or M2 macrophages were capable of being activated and fully functional, cells were either left untreated or treated with LPS/IFN−γ (0.1 µg/mL and 1 U/mL, respectively) for 24 h. Cells were stained with an anti-CD40 antibody and analyzed by flow cytometry. (A) FSC/SSC dot plots and histograms showing CD40 expression of one representative donor are shown (black filled = untreated; dotted line = LPS/IFN-γ treated). (B) Graphs demonstrating the mean fluorescence intensity (MFI) of CD40 expression are given. Untreated cells were normalized to 100 (black bars = untreated; white bars = LPS/IFN-γ treated; n = 8; performed in 2 independent experiments). Error bars indicate standard deviations. ** ≤ 0.01 (Wilcoxon signed-rank test). [file Image_2.tif]

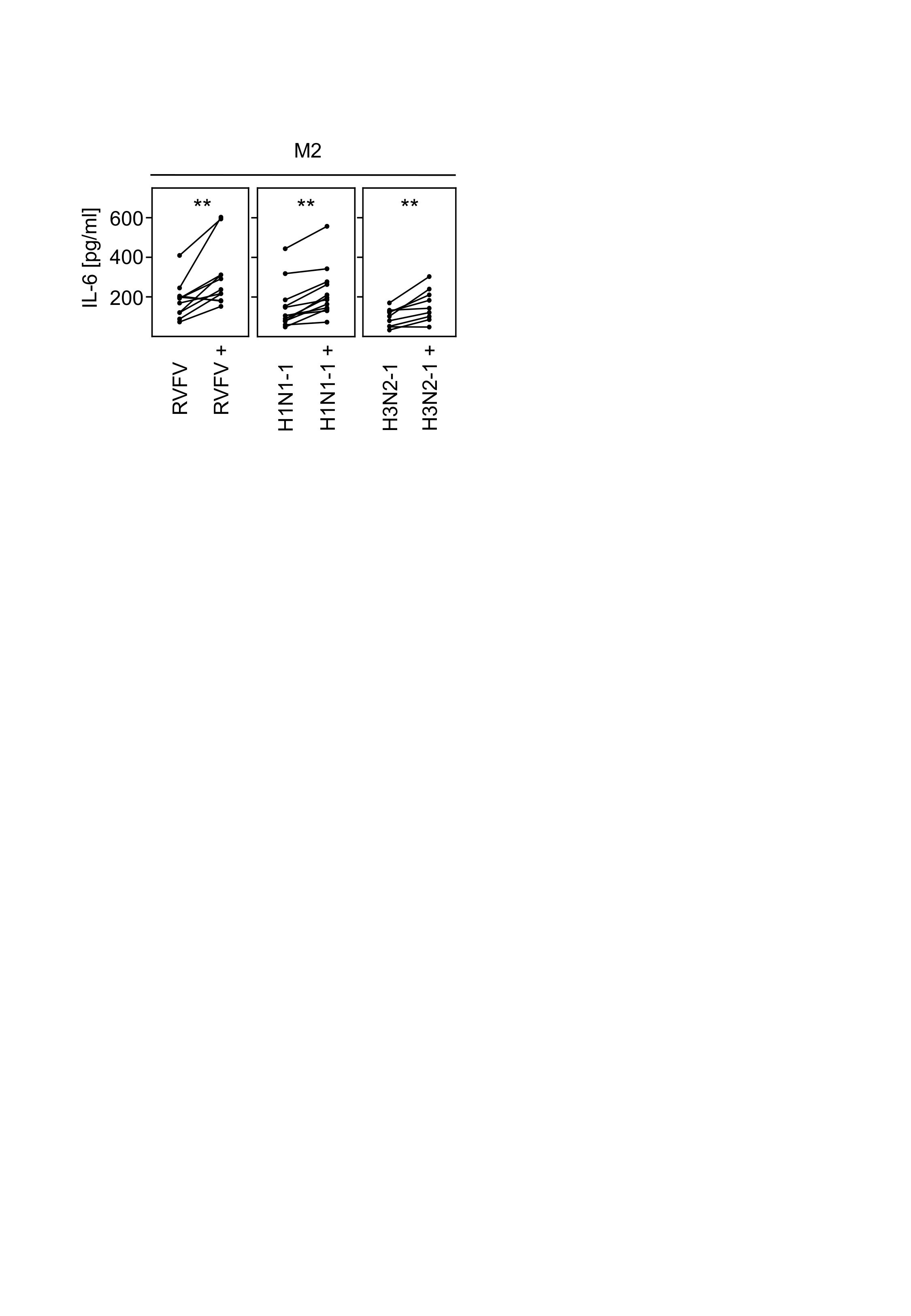

Supplement: Supplementary Figure 3 — Co-infection of SARS-CoV-2 synergistically increases virus-induced IL-6 production by M2 macrophages. Selected stimuli from Figure 5 are given in a before and after plot showing the IL-6 expression of M2 macrophages from each individual donor without and with SARS-CoV-2 co-infection (n = 9-12, performed in 2 experiment). Error bars indicate standard deviations. ** ≤ 0.01 (paired t-test). [file Image_3.tif]

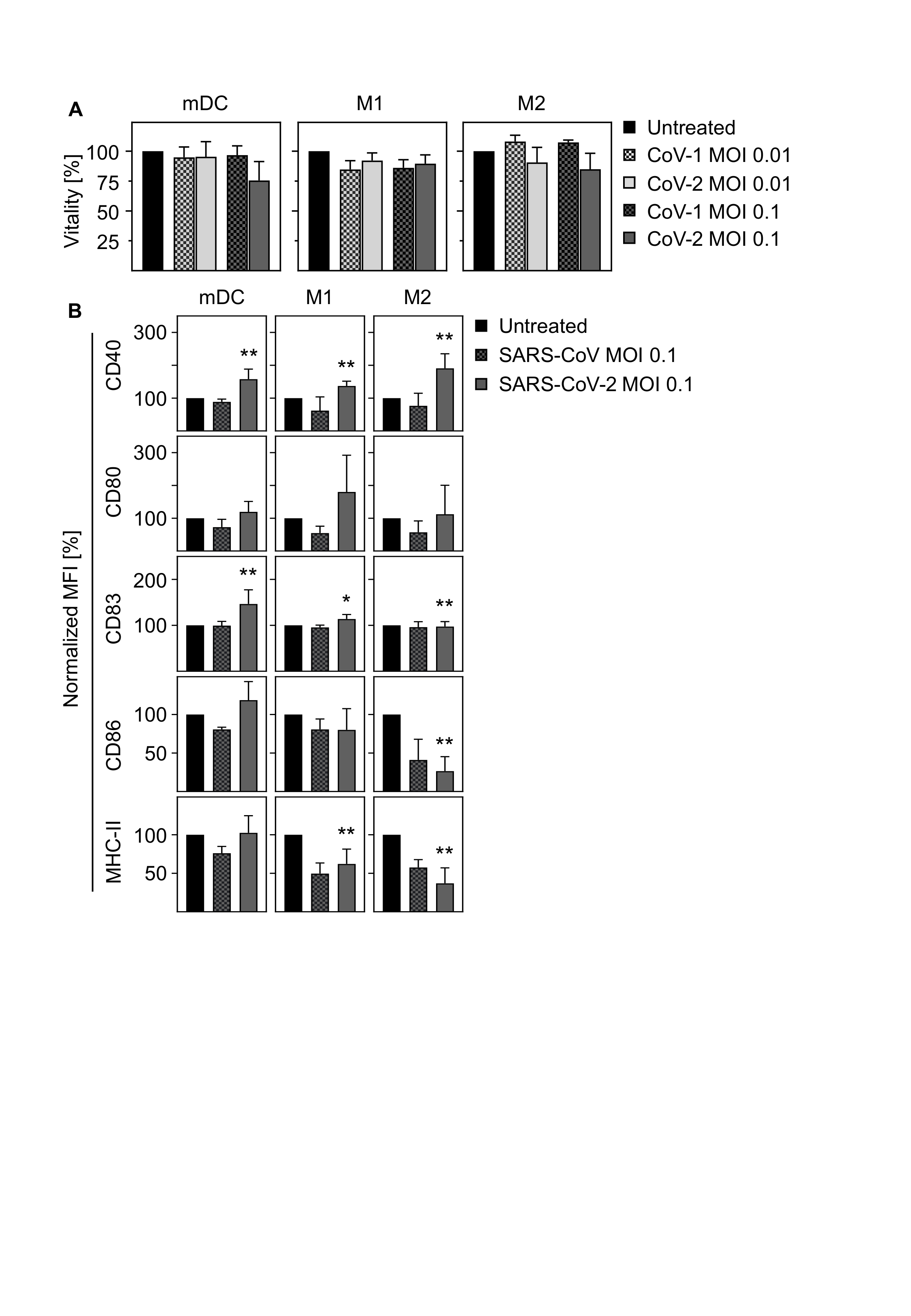

Supplement: Supplementary Figure 4 — Comparison of SARS-CoV-2 and SARS-CoV infected innate immune cells. (A) In vitro differentiated mDC, M1-, or M2 macrophages were infected with SARS-CoV (either MOI 0.01 or 0.1) and analyzed for viability by flow cytometry as described in Figure 1 . SARS-CoV-2 infected cells from Figure 1 are shown as comparison. Untreated cells (-) served as control. All viability data were normalized to untreated cells (n = 4-12; performed in 1-4 independent experiments). (B) mDC, M1-, and M2 macrophages were infected with SARS-CoV (MOI 0.1) for 24 h, stained for the activation/maturation markers CD40, CD80, CD83, CD86, and MHC-II, and analyzed by flow cytometry. Data for SARS-CoV-2 from Figure 3 are shown as comparison. Untreated cells (-) served as control. Bar graphs show MFI of the respective marker normalized to untreated cells (n = 3-8; performed in 1-2 independent experiments). Error bars indicate standard deviations. * ≤ 0.05; ** ≤ 0.01 (Wilcoxon signed-rank test). [file Image_4.tif]
